# The Multifaced Role of Collagen in Cancer Development and Progression

**DOI:** 10.3390/ijms252413523

**Published:** 2024-12-17

**Authors:** Gabriele Lo Buglio, Alessandra Lo Cicero, Simona Campora, Giulio Ghersi

**Affiliations:** 1 Department of Biological, Chemical and Pharmaceutical Sciences and Technologies (STEBICEF), University of Palermo, 90128 Palermo, Italy; gabriele.lobuglio@unipa.it (G.L.B.); simona.campora@unipa.it (S.C.); 2 Department of Pharmacy, University of Copenhagen, Universitetsparken 2, 2100 Copenhagen, Denmark; 3 Abiel srl, 90128 Palermo, Italy

**Keywords:** collagen, extracellular matrix, solid tumor, tumor microenvironment

## Abstract

Collagen is a crucial protein in the extracellular matrix (ECM) essential for preserving tissue architecture and supporting crucial cellular functions like proliferation and differentiation. There are twenty-eight identified types of collagen, which are further divided into different subgroups. This protein plays a critical role in regulating tissue homeostasis. However, in solid tumors, the balance can be disrupted, due to an abundance of collagen in the tumor microenvironment, which significantly affects tumor growth, cell invasion, and metastasis. It is important to investigate the specific types of collagens in cancer ECM and their distinct roles in tumor progression to comprehend their unique contribution to tumor behavior. The diverse pathophysiological functions of different collagen types in cancers illustrate collagen’s dual roles, offering potential therapeutic options and serving as prognostic markers.

## 1. Introduction

Collagen is the most abundant protein in the human body, accounting for 25–30% of its total protein content and is the main constituent of various tissues, including bones, ligaments, and skin [1]. As the fundamental component of the extracellular matrix (ECM), collagen plays a crucial role in the structural and functional organization of all tissues [2,3]. The ECM is a dynamic three-dimensional network of macromolecules, such as glycoproteins, proteoglycans, fibronectin, and laminin, with functions that are highly dependent on protein compositions. These functions include pH maintaining, regulating cell proliferation and differentiation, and providing support and anchorage for cells [4,5]. Collagen and other ECM components vary in structure and function, thereby regulating tissue homeostasis.

The equilibrium of ECM can be affected by injuries, aging, and various diseases [6]. Recent research studies have focused on understanding the role of ECM and collagen in the formation and progression of solid tumors. Together with tumor vasculature, associated connective tissues, infiltrating immune cells, and cancer-associated fibroblasts (CAFs), the component of ECM constitutes the tumor microenvironment (TME) of solid tumors [7]. Therefore, solid tumors are characterized by ECM rich in collagen, which encapsulates tumor cells, influencing cell accessibility and metabolism, and promoting tumor cell progression, invasion, and migration. This high collagen density leads to a TME stiffness that is significantly stronger compared to healthy tissues [8,9]. This condition is also influenced by the orientation of collagen fibers, which form a random reticle, dynamically remodeled during disease progression [10,11,12]. Excessive deposition and specific orientation of collagen are considered the major pathological features of solid tumors, allowing collagens to play active roles in cancer [13,14]. Increasing evidence suggests that the various types of collagens in the tumor ECM promote tumor proliferation and invasion and create conditions that enhance metastatic dissemination [15].

This review aims to provide an overview of the pathological role of different types of collagens in solid tumors, highlighting how these molecules represent excellent therapeutic targets and reliable prognostic markers. By exploring the diverse functions of collagen within the ECM, it is possible to better understand its impact on tumor biology and identify novel approaches to modulate the ECM for improved cancer treatment outcomes.

## 2. Collagen: Role and Structure

Along with fibronectin, laminin, and elastin, collagen constitutes the matrisome of the ECM, composed of approximately 300 proteins [16]. Collagen is a complex and dynamic macromolecule, assuming various structures in different tissues, conferring them a range of biological functions. To date, 28 collagen subtypes have been identified, with type I collagen representing over 90%, being the primary component of organs and connective tissues [6]. As a key component of connective tissues, collagen plays an essential role in maintaining their structural and biological integrity [17]. The synthesis and remodeling of collagens are dynamic mechanisms that are closely dependent on the physiological and, in some cases, pathological conditions of the tissue. These processes primarily involve fibroblasts, chondrocytes, osteoblasts, smooth muscle cells (SMCs), epithelial cells, endothelial cells, hepatic stellate cells, and cardiac myocytes [18,19,20,21,22,23,24]. The α chains of various types of collagen are used to build trimeric molecules, which intertwine into a 300 nm long triple helix and subsequently assemble into fibrils. Some types, such as type II collagen, form homotrimeric molecules, while others, such as types I and V, form heterotrimers [25,26]. The collagen components interact in a specific sequence with each other and with other tissue proteins and cells, producing higher-order structures. For example, continuous crosstalk occurs between collagen fibers and resident fibroblasts, allowing for a balance of tensile and recoil energy in a tissue, and modulating mechanotransduction pathways [27]. The modulation of the intracellular signaling pathways also depends on the turnover of the various collagens within the extracellular matrix. This process is highly dependent on the activity of specific proteases, particularly matrix metalloproteinases (MMPs) secreted by resident cells [28,29]. These enzymes modify the different extracellular matrix components, altering the density of the microenvironment and leading to mechanical loads perceived by the cells. Several studies have examined the influence of mechanical force on the extracellular matrix formation. However, the complexity of controlling and assembling collagen fibrils is still not well understood. Some studies suggest both cell-dependent and cell-independent models for collagen organization, highlighting the importance of mechanochemistry in determining fibril arrangement under mechanical stress [30].

## 3. Biosynthesis

The different types of collagen share the canonical sequence domain Gly-X-Y, where X and Y can vary with a consistent presence of proline and hydroxyproline residues [31]. Hydroxyproline is an amino acid not commonly found in the amino acid sequence of proteins; however, it may account for more than 50% of the total amino acid content of collagen [32]. The high percentage of these residues is critical for reducing the entropic contribution during collagen filament folding [33]. Collagen synthesis is a multi-step process involving several enzymes. After synthesis, pre-collagen undergoes disulfide bond formation at the N- and C-terminal propeptide regions followed by post-translational modifications, mainly on lysines and prolines, such as hydroxylation (via lysyl hydroxylase activity) and glycosylation (through the addition of glucose and galactose oligosaccharides), leading to the formation of pro-collagen α chains [34]. Lysine hydroxylation induces the formation of triple helices, which are then secreted into the extracellular environment via Golgi vesicles [15]. Once secreted, collagen undergoes a combined activity of lysyl oxidases (LOX) and some classes of LOX-like proteins (LOXL) [35], which function as cross-linking agents found both in the intracellular and extracellular environment. They are necessary for collagen’s mechanical properties acquisition, such as tensile strength, contributing to the regulation of ECM stiffness [36,37].

## 4. Tumoral Extracellular Matrix

**Figure 1:**
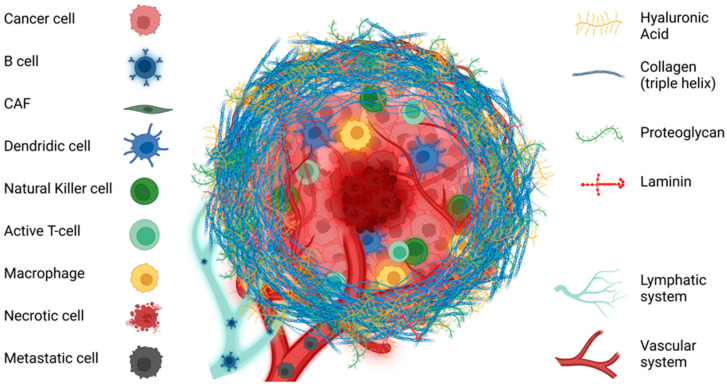
Schematic representation of the major components of tumor microenvironment in solid tumors. The tumor microenvironment mainly consists of tumor cells, immune cells, stromal cells, and extracellular matrix. The primary factor contributing to matrix stiffness is the excessive presence of collagen, which significantly impacts various crucial biological processes throughout tumor development.

Tumorigenesis is a complex and dynamic process involving the cooperation of various elements within the tumor microenvironment (TME) [7]. The TME components play several critical roles in promoting and sustaining tumor progression. These roles include the deregulation of proliferative signals and the evasion of growth suppression control. Consequently, tumor cells acquire several characteristics: resistance to apoptosis, uncontrolled proliferation, promotion of tissue colonization, modulation of cellular metabolism, stimulation of angiogenesis, and evasion from the immune system [38]. The TME is composed of multiple cellular components, such as endothelial cells (ECs), immunocytes, adipocytes, cancer-associated fibroblasts (CAFs), and the ECM [3] (Figure 1).

CAFs are pivotal components of the TME with enhanced functional properties, such as proliferation, migration, and secretion. They persist in a hyperactivated condition and exhibit elevated metabolic activity. Their activity contributes to shaping a thick tumoral extracellular matrix, through synthesis and abnormal deposition of fibronectin, laminin, hyaluronic acid, and collagen types I, III, IV, and V, above all collagen type I [39,40,41].

The ECM plays a key role in the TME, encapsulating tumor cells within an acidic and hypoxic extracellular matrix, which reduces cell accessibility and influences their metabolism. From a structural point of view, the ECM of solid tumors is an envelope that surrounds, protects, and interacts with tumor cells. Moreover, due to its macromolecular composition, the tumor ECM exhibits abnormal rigidity compared to the ECM of healthy tissues. For example, while the stiffness of the ECM in healthy tissues such as the breast, brain, liver, lung, and pancreas is typically around 1000 Pa, it can reach values between 4 and 10 kPa in tumors located in these same sites [8]. This increased stiffness can result from different biomechanical factors, including an imbalance between matrix deposition and degradation, the presence of contractile actomyosin in CAFs, interactions between tumor and normal cells, and especially altered collagen cross-linking [42,43]. Tumor ECM shares several components with healthy tissues ECM, such as collagens, elastin, proteoglycans, and glycoproteins like tenascin-C, laminins, and fibronectins [44]. However, changes in the relative composition of these elements lead to structural and functional alterations that promote tumor development [45]. The ECM components contribute to a fibrotic extracellular environment, modified by multiple factors, such as MMPs. MMPs actively contribute to tumor progression by participating in ECM turnover, which influences tumor cell proliferation, angiogenesis, and invasion, as well as apoptosis inhibition [46].

## 5. Collagens in Solid Tumors

Collagen is a key component of the ECM in the TME, significantly contributing to tumors’ physical and biochemical properties. The organization and density of collagen fibers can significantly affect the progression and spread of tumors. Collagen provides structural support to tumors, creating a scaffold that aids tumor growth. Increased collagen deposition leads to a stiffer ECM, promoting tumor cell proliferation and survival. Cancer cells interact with collagen through integrins and other receptors, transducing signals that influence tumor cell behavior, leading to cell growth, invasion, resistance to chemotherapy, metabolism variation, invasion, migration, and evasion to the immune system.

### 5.1. Collagen and Cancer Cell Proliferation

Collagen and other ECM components interact directly with tumor cells, influencing their growth. These molecules play an important role in regulating the cell cycle. Some fibrillar collagens suppress tumor growth by interacting with Discoidin Domain Receptor 2 (DDR2), a transmembrane receptor tyrosine kinase (RTK). Along with Discoidin Domain Receptor 1 (DDR1), DDR2 binds collagen in the extracellular matrix of various tissues. This interaction leads to cell cycle arrest at the G1/S checkpoint [47]. Other collagens, such as type III collagen, induce tumor cells into dormancy via the DDR1/Signal Transducer and Activator of Transcription 1 (STAT1) signaling pathway [48]. However, most of them promote tumor cell proliferation through various pathways. For instance, it has been shown that monomeric collagen can inhibit the expression of p21 in tumor cells in vitro, altering cell cycle restrictions [49]. Moreover, collagen interaction with integrin beta-1 leads to increased tumor cell proliferation, through the activation of β-catenin [50,51] However, collagen regulates tumor cell proliferation through several mechanisms, including diverse metabolic pathways.

### 5.2. Collagen and Tumor Metabolism

One of the aspects that over the years led to significant attention in the scientific community is tumor glucose metabolism. Tumor cells can absorb more glucose than their healthy tissue counterparts, converting it into lactate [52]. This process, known as the Warburg effect, is regulated by numerous factors, including matrix collagen. It has been demonstrated in vitro that increased collagen density promotes aerobic glycolysis in human breast cancer cell lines, stimulating integrin activation [53]. This activates the Posphoinositide 3-Kinase (PI3K)/Protein Kinase B (Akt) signaling pathway, which positively modulates the activity of various factors upstream, such as Glucose Transporter 1 (GLUT1) and pyruvate kinase M2 (PKM2) [54,55]. Furthermore, these metabolic anomalies modify glutamine uptake and its fate in tumors. In this context, a recent in silico study focused on the increased glutamine uptake in tumor cells exploiting the Warburg effect, noting that this metabolite is directed toward stromal collagen production.

Thus, through positive feedback mechanisms, tumor cells link collagen with glucose and lipid metabolism, promoting tumor progression [56,57,58].

### 5.3. Collagen and Apoptosis

Apoptosis represents a highly regulated cell death process governed by various endogenous or exogenous stimuli. However, in the tumorigenesis contest, this mechanism is completely dysregulated, promoting tumor growth at the expense of surrounding tissues [59]. The involvement of matrix collagen in anti-apoptotic processes is not fully understood. The presence of collagen in gastric cancer has been shown to upregulate, both in vitro and in vivo, the expression of anti-apoptotic factors, such as B-cell lymphoma 2 (BCL2), increased by the β-catenin pathway [60]. Moreover, DDR1 pathway activation mediated by type I collagen inhibits tumor cell growth, through the pro-apoptotic factor BCL-2 interacting killer (BIK), a member of the BCL2 family [61,62]. Collagen can also suppress tumor growth by negatively modulating certain cell cycle factors, such as p21 and CDKs, and regulating caspase expression [63]. In addition to collagen, some of its peptide derivatives can induce apoptosis, by Bcl-2 and BCL-extra large (BCLxL) pathways [64].

To resume, the dual role of collagen raises many questions about its actual function in solid tumors. Given its pleiotropic effects on malignancy, different factors may play crucial roles, such as tumor type, staging, and the specific type of matrix collagen.

### 5.4. Collagen and Tumor Cell Invasion

Among the hallmarks of solid tumors, the invasion of tumor cells is one of the most determining factors for patient prognosis since this mechanism plays a crucial role in tumor progression and metastasis formation [65]. For instance, collagen regulates the extension of invadopodia through the integrin αvβ3/Dishevelled-associated activator of morphogenesis 1 (DAAM1)/Ras Homolog family member A (RHOA)-dependent cascade [66], which are small actin-rich protrusions that mediate the invasion of tumor cells [67]. From a biophysical perspective, the increased stromal tension caused by matrix collagen accumulation in breast cancers enhances the integrin β1-mediated signaling pathway, leading to the activation of the Focal Adhesion Kinase (FAK)/Yes-Associated Protein (YAP) pathway [68]. Activation of integrin β1 can be triggered by mutated collagens at one or more sites, which accelerate the tumor cell invasion, as observed in invasive epithelial tumors [69]. However, YAP activation must be corroborated by collagen matrix remodeling, mediated by Lox3 and MMPs, which are regulated by the microenvironment stiffness [70]. On the other hand, collagen degradation, or the loss of ECM stiffness induced by ultraviolet radiation (UVR) against fibroblasts, could surprisingly increase the survival of melanoma patients due to the lack of matrix support for invasiveness [71].

### 5.5. Collagen and Metastasis

Besides driving tumors to cell invasion, collagen plays a key role in cell migration. The ECM of solid tumors changes the acquisition of metastatic potential [72], and the collagen within it acts as a “molecular binary” to increase the tumor cell’s escape from the primary site [73,74]. However, cancer research has recently focused on the pathways regulated by stromal collagen implicated in this process. For instance, the DDR1 or DDR2 activation mediated by collagen positively regulates the metastatic process involving tumor-associated neutrophils (TANs) and CAFs [75,76]. Upregulation of collagen prolyl-4-hydroxylases (P4HA) of class I and II, whose expression is increased by tissue hypoxia [77], could drive collagen deposition in metastatic ovarian tumors, leading to neo-angiogenesis and tumor cell migration [78].

Thus, metastasis is a complex process involving multiple converging and diverging signaling pathways, often upstream regulated by collagen. Some pathways are even independent, such as versican (VCAN)/Extracellular signal-Regulated Kinase (ERK) and poly [ADP-ribose] polymerase 1 (PARP1)/Zinc finger E-box-Binding homeobox 1 (ZEB1) pathways, which promote the proliferation and migration of colorectal cancer cells, regulated directly or indirectly by collagen [79]. In other cases, collagen can be secreted into exosomes to promote metastasis, as observed in osteosarcomas [80].

### 5.6. Collagen and Tumor Angiogenesis

Increased collagen deposition and its alignment are characteristics that impact neovascularization [81,82]. More specifically, the high density of collagen and other matrix components creates a hypoxic environment at the tumor core, activating the hypoxia-inducible factor (HIF-1) [83]. HIF-1 is a pivotal transcription factor for vascular endothelial growth factor (VEGF), which stimulates neo-angiogenesis and actively contributes to tumor progression [84]. Additionally, HIF-1 is involved in immune evasion mechanisms through the expression of immunosuppressors, such as those regulating antigen presentation to Natural Killer (NK) cells [85].

Thus, collagen plays a physical role in tumor vascular system formation. However, some studies have also examined the biochemical role of collagen in neo-angiogenesis, investigating the pathways in which this protein is involved. More specifically, some of its peptide derivatives exhibit anti-angiogenic effects involving integrins αvβ3 and α5β1 [86]. Other peptides, including those derived from type IV collagen, inhibit endothelial cell proliferation stimulated by basic fibroblast growth factor (bFGF) or induce apoptosis in endothelial cells via a Fas-dependent mechanism [87], confirming the pleiotropic nature of tumor-associated collagen.

### 5.7. Collagen and Chemoresistance

Despite the significant advancements in research, chemoresistance remains the main challenge in tumor relapse and therapeutic ineffectiveness [88]. ECM, particularly collagen, plays a crucial role in chemoresistance, acting as a physical barrier against drugs. Additionally, collagen can influence various biochemical processes, such as dormancy, where tumor cells enter a quiescent state and become desensitized to chemotherapeutic agents before re-entering the G1/S phase [89,90]. Another important mechanism of chemoresistance is drug efflux, mediated by the upregulation of the ATP-binding cassette (ABC) superfamily, with P-glycoprotein (ABCB1) being the most well-known factor [91]. Specifically, ABCs can be positively modulated by α2β1-integrin, which is activated by the stromal collagen [92]. Moreover, collagen can also affect the transcription of certain genes to promote chemoresistance. In ovarian carcinoma, collagen can stabilize and activate DNA methyltransferase 1 (DNMT1), which leads to methylation and downregulation of miR-509-3p promoter, an anti-tumor miRNA that also suppresses chemoresistance [93].

Therefore, in addition to physically preventing drug entry, collagen in the tumor matrix can access multiple signaling pathways that reduce therapeutic efficacy.

### 5.8. Collagen and Immunomodulation

ECM plays an immunomodulatory role in solid tumors. This dense and fibrotic structure, rich in collagen, creates barriers that hinder the infiltration of chemotherapeutics and immune cells. For instance, the ECM can influence leukocyte localization and transmigration, reducing their accessibility to the tumor mass [94,95]. Among the matrix components, collagen regulates immune cell activity, such as T lymphocytes and macrophages, often favoring tumor progression [96,97]. The increased matrix density and collagen alignment suppress the tumor-infiltrating T lymphocyte (TIL) activity [98]. It has been shown in vivo that tumor collagen alignment mediated by the DDR1 extracellular domain binding hinders the infiltration of CD4+ and CD8+ T lymphocytes [99]. Supporting this study, the use of PRTH-101 (monoclonal anti-DDR1 antibodies) in breast cancer mouse models disrupted stromal collagen organization, increasing immune infiltration and, consequently, survival [100]. Beyond DDRs, collagen has receptors on leukocytes, such as leukocyte-associated immunoglobulin-like receptor 1 (LAIR-1). This receptor has an essential physiological role in preventing the immune system’s uncontrolled responses, such as autoimmune reactions [101]. However, when LAIR-1 binds collagen of the tumor microenvironment, the antitumor potential of cytotoxic T lymphocytes is suppressed in an SHP-1-dependent pathway, as seen in gliomas [102]. Therefore, LAIR-1 has recently become a therapeutic target to counteract immune evasion in solid tumors [103,104]. Similarly, collagen fragments generated by MMP1 and MMP9 can bind LAIR-1, leading to immune suppression. LAIR-2, a soluble homolog of LAIR-1, reverses T lymphocyte collagen-mediated suppression through a competitive mechanism [105].

Thus, tumor collagen and the other associated factors, such as lysyl oxidase, represent important therapeutic targets to enhance immune responses against solid tumors [106,107,108,109,110]. However, some cytokines, such as chemokines produced by immune cells, can contribute to tumor matrix formation or even interfere with the antitumor activity of the immune system. Indeed, specific interleukins (IL), such as IL-4, IL-6, and IL-13, induce collagen synthesis, contributing to tissue fibrosis and tumor progression. In addition, other factors, such as IL-10 and Transforming Growth Factor Beta-1 (TGF-β), secreted by M2 macrophages, inhibit Th1 lymphocyte responses and immune cell activation, creating an environment that supports tumor growth and immune evasion [111,112]. Moreover, it has been studied that the collagen I/DDR1 axis activates the Protein Kinase C theta (PKCθ)/Spleen tyrosine kinase (SYK)/Nuclear Factor kappa-light-chain-enhancer of activated B cells (NF-κB) cascade, resulting in the upregulation of the chemokine C-X-C motif chemokine 5 (CXCL5). This chemokine leads to the formation of neutrophils extracellular traps (NETs) by TANs, a key mechanism driving the metastatic process [113,114].

Therefore, the relationship between the immune system and the stromal collagen in solid tumors is ambiguous, where even some immune cells can actively participate in the ECM formation and tumor progression. A striking example is tumor-associated macrophages (TAMs). These macrophages acquire specific phenotypic characteristics, polarizing from antitumoral (M1) to protumoral (M2) states through a mechanism induced by IL-4 secreted by CD4+ T cells [75]. These cells influence the tumor progression, enhancing neo-angiogenesis or tumor cell migration [115,116]. Additionally, TAM2s can secrete collagen I to activate the α2β1 integrin/PI3K/Akt axis, supporting tumor progression [117,118].

For this reason, continued studies on the relationship between stromal collagen and the immune system may uncover targeted therapies against solid tumors.

## 6. Mechanotransduction Mediated by Collagen in Cancer

Mechanotransduction is a complex process in which the mechanical properties of a tissue modulate intracellular signaling pathways, essential for the homeostasis of its constituent cells [119]. Mechanical homeostasis is regulated by the deposition and orientation of matrix components, such as collagens and fibronectin. The balance of stretching and tensile forces determines the elasticity or stiffness of the microenvironment. In this contest, resident cells can perceive the stiffness by acting as mechanosensors [120]. Moreover, focal adhesions are fundamental to the mechanical stimuli reception, mediating cell–matrix contacts in synergy with specific linker proteins, such as integrins and actin cytoskeleton components [121,122]. Additionally, the modulation of mechanical stimuli within the tissue microenvironment can be influenced by the cells, neighboring tissues, or the hydrostatic pressures of surrounding fluids [123]. Within this context, signaling pathways arising from the intricate relationship between a tissue’s biochemistry and physical properties regulate gene expression, modulating the phenotype and primary metabolic activities of resident cells, including proliferation, differentiation, migration, and programmed cell death mechanisms [124]. However, mechanotransduction is also fundamental in the pathology of various diseases. For instance, in tumors, the heterogeneous increase in density and cross-linking of ECM components leads to abnormal stiffness compared to healthy tissue [125]. Generally, receptor tyrosine kinases (RTKs), such as AXL receptor tyrosine kinase (AXL), Receptor Tyrosine Kinase Like Orphan Receptor 2 (ROR2), and ephrin receptors (EPHs), detect matrix contractions across different hotspot regions, working as mechanosensors [126]. For instance, it has been demonstrated that EPHA2, in the absence of its canonical ligand and in cooperation with integrin beta-1, senses the stiffening of the tumor microenvironment. This detection activates the Lck/yes-related protein tyrosine kinase (LYN) signaling pathway, resulting in the phosphorylation and nuclear translocation of the transcription cofactor Twist-related protein 1 (TWIST1). Consequently, the activated pathway triggers the epithelial–mesenchymal transition (EMT) required for tumor cell invasion [127].

The interactions in the cell–matrix interface between cancer cells and collagen can activate the transcriptional coactivator YAP. YAP selectively reprograms tumor cells, promoting the transition of outer basal tumor cells into leader cells with invasive capacities. Furthermore, YAP activation enhances the transcription of ECM remodelers involved in collagen cross-linking, establishing a positive feedback mechanism that amplifies the invasive potential of tumor cells [128,129]. Different studies have highlighted that YAP nuclear translocation is facilitated by morphological modifications of nuclear pores, induced by increased stress fibers in the perinuclear region connected to the tumor ECM [130,131,132].

Mechanical signals from the tumor microenvironment also amplify tumor cell proliferation through positive feedback mechanisms. For instance, excessive collagen deposition and other tumor matrix components are physically sensed by stromal mesenchymal stem cells (MSCs). In response, MSCs start to secrete the protein prosaposin (PSAP), which promotes tumor growth via activation of the Toll-Like Receptor (TLR)/NF-κB signaling pathway [133,134,135]. Additionally, matrix stiffness drives the differentiation of MSCs into cancer-associated fibroblasts (CAFs), which are primary contributors to collagen secretion, further reinforcing overall matrix stiffness in a self-perpetuating cycle [136].

Beyond cell invasiveness and proliferation, continuous remodeling and stiffening of the tumor-associated extracellular collagen activate angiogenic signaling necessary for new vessel development. This process engages the integrin β1/PI3K/Akt signaling pathway, promoting the secretion of factors such as activin A, amphiregulin, artemin, interleukin (IL-1β), persephin, urokinase-type plasminogen activator (uPA), and vascular endothelial growth factor (VEGF) [127,137,138]. Thus, mechanotransduction triggered by matrix components, specifically collagen, underlies cancer pathology by driving processes such as differentiation, proliferation, invasion, and angiogenesis.

## 7. Collagen Classification

**Figure 2:**
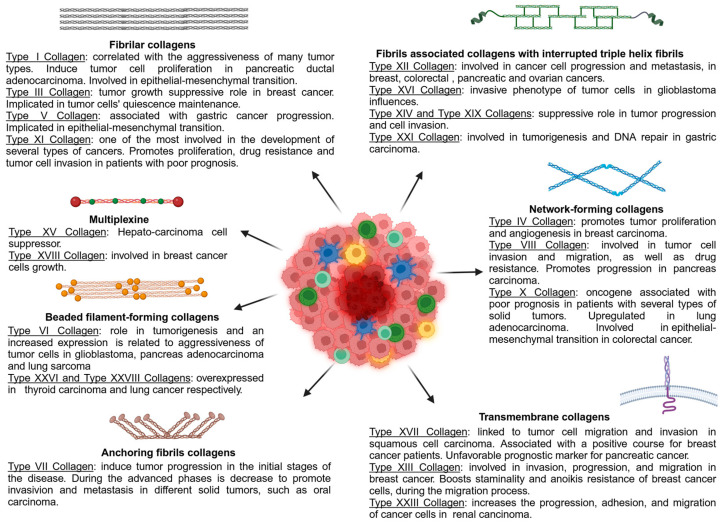
Classification of collagens based on supramolecular assembly and a schematic overview of the major roles of each collagen type in tumorigenesis.

The collagen superfamily is characterized by twenty-eight types of collagens, which are further divided into different subgroups [139]. In vertebrates, collagens are numbered using Roman numerals (I–XXVIII) [140] and are classified into fibrillar and non-fibrillar types. The predominant category is represented by fibrillar collagens, which constitute 90% of the total. These fibrillar collagens have elongated fibril-like structures (rods or bands) and include types I, II, III, V, XI, XXIV, and XXVII. On the other hand, non-fibrillar collagens form various supramolecular structures and are divided into different categories, including fibrils-associated collagens with interrupted triple helices (FACITs) (types IX, XII, XIV, XVI, XIX, XX, XXI, and XXII), network-forming collagens (types IV, VIII, and X), beaded filament-forming collagens (types VI, XXVI, and XXVIII), anchoring fibrils (type VII), transmembrane collagens (types XIII, XVII, XXIII, and XXV), and multiplexins (types XV and XVIII) [141,142]. Various collagens contribute uniquely to the tumor microenvironment, affecting tumor behavior and metastasis through different pathways, such as mechanotransduction (Figure 2).

### 7.1. Fibrillar Collagens

Fibrillar collagens have a continuous triple-helix structure of approximately 300 nm ø, followed by a chain of 1000 amino acids. This group includes types I, II, III, V, XI, XXIV, and XXVII collagens, which promote the development of solid tumors [143]. Several studies have analyzed collagen fibers’ architecture, characteristic of the solid tumor ECM. While it is well established that tissue fibrosis precedes neoplasms, recent findings increasingly confirm that the stratification and elongation of stromal fibers, particularly their orientation, are statistically associated with a highly aggressive tumor cell phenotype [144]. This is because the orientation of collagen fibers in the tumor ECM differs from that of healthy tissues. Specifically, while in healthy tissues collagen is isotropically oriented, tumor tissues often exhibit an anisotropic orientation, mainly due to an increased ratio of type I to type III collagen [145]. Type I collagen is not only the most abundant protein in the body, but it is also prevalent in solid tumors. Increased deposition of type I collagen (COL1) in the tumor ECM is closely correlated with the aggressiveness of many tumor types, such as triple-negative breast carcinoma, pancreatic ductal adenocarcinoma (PDAC), or gastric carcinoma [146]. For example, COL1 induces cells to spread and reorganize their cytoskeleton in gastric carcinoma, promoting metastasis. This occurs because COL1 decreases adhesion between cells by disassembling the E-cadherin/catenin complex, which is known to be involved in the process of cell adhesion [147,148]. Furthermore, in pancreatic ductal adenocarcinoma, COL1 interacts with Discoidin Domain Receptor 1 (DDR1) [149]. When active, DDRs trigger cellular signals in tumors that affect various functions, including extracellular matrix homeostasis, tumor cell proliferation, and migration [150]. For instance, when DDR1 binds COLI, the proliferation and invasion of tumor cells are promoted through the activation of FAK-related protein tyrosine kinase 2 (PYK2), which consequently downstream activates the Crk-associated substrate (CAS)/Ras-Proximate-1 (RAP1)/c-Jun N-terminal Kinases (JNK1)/c-Jun cascade. Moreover, the cleavage of COL1 by MMPs differently affects the bioenergetics and growth of pancreatic ductal adenocarcinoma via DDR1: matrix-metalloprotease-cleaved COL1 (cCOL1) activates a signaling cascade that promotes tumor growth, while intact COL1 (iCOL1) induces DDR1 degradation, resulting in opposite effects. Patients with enriched iCOL1 exhibit better survival compared to those with tumors enriched in cCOL1 and DDR1, suggesting that targeting the DDR1/NF-κB/Nuclear Factor Erythroid 2-Related factor 2 (NRF2)/Mitochondrial Transcription Factor A (TFAM) pathway could have therapeutic benefits [151]. In addition, COL1 is implicated in the promotion of epithelial–mesenchymal transition (EMT), a key process in embryonic development characterized by the loss of epithelial markers by epithelial cells [152]. Although typically inactive in adults, EMT has been associated with tumor invasion and metastasis. COL1-mediated phosphorylation of IκB is one pathway through which EMT is activated, leading to increased expression of the transcription factors Snail and Lymphoid Enhancer Binding Factor 1 (LEF-1), which decreases the expression of E-cadherin [153]. In addition, COL1 may be a rich source of exploitable metabolic fuel for cancer cells under critical nutrient-deficient conditions [154] and actively involved in tumor progression and metastasis. However, the role of type I collagen produced by CAFs can sometimes be shown to be contradictory since it can also have antitumor activity. The mechanisms underlying these opposing actions remain not entirely clear [139,155].

In contrast to type I collagen, type III collagen (COL3) has been shown to potentially play a tumor growth suppressive role [146]. For instance, in breast cancer patients, COL3 protects the tissues and organs of affected patients, and its reduction enhances the invasiveness of tumor cells. For example, in mice xenografted with breast cancer cells, COL3 inhibited tumor growth [156]. This is because type III collagen is implicated in tumor cells’ quiescence (G0 phase) maintenance. The re-entry of quiescence cancer cells into the cell cycle plays an important role in cancer recurrence. A reduction in COL3 leads not only to cell proliferation but also amplifies the metastatic potential of cancer cells, through modulation of the DDR1/STAT1 pathway [48]. However, the concept of quiescence in cancer remains a matter of debate. Indeed, although a state of quiescence might seemingly be a tumor progression obstacle, some studies claim that in some cases this would represent a defense mechanism from drugs. For example, cancer cells stressed by the presence of drugs enter a long persistence phase, where they can acquire chemoresistance. This drug-tolerance state is reversible as dormant tumor cells can reactivate their proliferation after drug removal; however, this is a process that increases sensitivity [157]. A further study reported that a type III humanized recombinant collagen (rhCOLIII) inhibited breast cancer cell proliferation and invasion in vitro, as well as promoting apoptosis. In addition, rhCOLIII treatment increased DDR1 protein expression, inhibiting autophagy of tumor cells [158]. This is because autophagy regulates cancer stem cell properties and contributes to the maintenance of stemness, development of relapse, and drug resistance [159].

Similar to type I collagen, type V collagen (COL5) created great interest in the scientific community, due to its involvement in tumorigenesis. In particular, type V collagen alpha 2 (COL5A2) has been observed to be associated with gastric cancer progression since this was found to be upregulated in affected patients. COL5A2 has been shown to play a direct role in cell proliferation of gastric cancer cells, where triggering the FAK/PI3K/Akt/mammalian Target Of Rapamycin (mTOR) signaling pathway [160,161]. It is also implicated in epithelial–mesenchymal transition in gastric cancer patients with poor prognosis, by promoting the expression of mesenchymal markers (SNAI1, SNAI2, TWIST, VIM, and MMP2) [162].

Among fibrillar collagens, type XI collagen (COL11) is one of the most involved in the development of several types of cancers, such as papillary thyroid carcinoma, breast cancer, colorectal carcinoma, esophageal cancer, gastric cancer, pancreatic cancer, lung cancer, and ovarian cancer [63,163,164,165,166,167,168,169]. Misregulated secretion of COL11 by CAFs creates a crosstalk between tumor cells and the TME, promoting proliferation, drug resistance, and tumor cell invasion in patients with poor prognosis [170,171,172]. In this context, it has been analyzed that integrin α1β1 and DDR2 act as extracellular Collagen 11 Alpha 1 Chain (COL11A1) receptors, triggering some signaling pathways related to ovarian cancer progression, such as Akt/CCAAT-Enhancer-Binding Proteins (C/EBPβ)/Phosphoinositide-Dependent Kinase-1 (PDK1) [173,174]. Additionally, in mouse models of xenograft with ovarian cancer cells, COL11A1 has been shown to stimulate IL-6 production via induction of TGF-β3, modulating the interaction between CAFs and tumor cells and amplifying tumor invasiveness and progression [172].

A recent study focused on the role of the gene that encodes type XXVII collagen, rather than the role of the collagen itself. Specifically, through an in vitro and in vivo analysis, the study examined the role of a specific miRNA (miRNA-455-3p) located on the gene encoding intron of Alpha Chain 1 of type XXVII collagen (COL271A). Notably, it was observed that in melanoma cells, this miRNA is closely involved in tumor cell dormancy, and its downregulation increased the re-entry of quiescent tumor cells into the cell cycle [175].

### 7.2. Fibrils-Associated Collagens with Interrupted Triple Helices (FACITs)

Among nonfibrillar collagens, collagens associated with interrupted triple-helix fibrils (FACITs) represent a group of small leucine-rich proteoglycans involved in regulating fibril formation [176]. Unlike fibrillar collagens that form long fibers, FACITs have interruptions in their triple-helix structure. These discontinuities are due to the presence of domains within the collagen molecule that can vary in length and composition, providing binding sites for other components of the extracellular matrix, such as fibrillar collagens [177,178]. Some collagens, such as types XII and XIV, are typically involved in stabilizing the extracellular matrix fibrils, working synergistically with type I. However, the specific organization is disrupted in the tumor microenvironment, resulting in a fibrotic and heterogeneous architecture that plays a functional role in tumorigenesis [59,100,101]. For this reason, several studies have been carried out on the distinct genes encoding for cancer-involved FACITs. For instance, type XII collagen alpha 1 chain (COL12A1) is implicated in cancer cell progression and metastasis [179] and it has been identified as a potential marker of poor prognosis in patients with breast cancer, colorectal cancer, pancreatic adenocarcinoma, and ovarian cancer. Furthermore, recent studies of temporal changes in breast cancer matrisome profile suggest that high levels of collagen XII in primary tumor ECM can facilitate the spread of lung cancer cells [180]. COL12A1 upregulation has also been correlated with metastasis, such as lymph nodes, and reduced patients’ survival with human epidermal growth factor receptor 2 (HER2)-positive breast cancer [181]. Moreover, COL12A1 expression in colorectal cancer has been linked with the regulation of several pathways, such as focal adhesion, and the PI3K-Akt pathway [182]. In patients with poor prognosis pancreatic adenocarcinoma, W. Yao et al. analyzed the relationship between COL12A1 and cytoplasmic poly (A) binding protein-1 (PABPC1), a class of RNA-binding proteins known to modulate tumor progression. PABPC1 acts as an oncogene, accelerating the proliferation and metastasis of human pancreatic cancer cell line BXPC3 through the upregulation of COL12A1 expression [183].

Type XVI Collagen (COL16) has been observed to be upregulated in certain tumor types, such as colorectal cancer (CRC), glioblastoma, and in some dysplastic areas of the mucosal epithelium of patients with oral squamous cell carcinoma [184,185]. In glioblastoma, Col16 expression influences the integrin β1 activation pattern, increasing the invasive phenotype of tumor cells [186].

Vice versa, type XIV (COL14) and type XIX (COL19) collagens exert a suppressive role in tumor progression and cell invasion. Indeed, in MMP14Sf-/- mice, the accumulation of Col14 in peritumoral areas of melanoma is correlated with a reduction in malignancy [187]. Similarly, the NC1 domain of Col19, inhibits tumor cell migration and invasion, as seen on SK-MEL-28 human melanoma cells. NC1 inhibits the FAK/PI3K/Akt/mTOR pathway, reducing the activity of proteins involved in transduction [188].

Information regarding the impact of type XX, XXI, and XXII collagens in tumors is limited. However, recent studies have shown that pancreatic ductal adenocarcinoma patients present elevated levels of circulating type XX and type XXII collagens in their serum, suggesting a possible prognostic value [189]. Another study focused on identifying hub genes and pathways in gastric carcinoma development, found that type XXI collagen alpha chain 1 (Col21a1) might have a direct role in the enrichment of some pathways involved in tumorigenesis, such as DNA repair and KRAS signaling pathway [190].

### 7.3. Network-Forming Collagens

Among the network-forming collagens, type IV collagen is the most prevalent in basement membranes, is approximately 400 nm in length, and assembles irregularly when forming networks. In contrast, type VIII and X collagens are shorter and form more regular hexagonal networks [191]. The expression mechanisms of network-forming collagens can be regulated by several transcription factors and play a crucial role in the development of solid tumors [143]. Type IV collagen has recently been studied in the ECM of several solid tumors. In mammals, there are six homologous α type IV collagen chains (α1(IV)–α6(IV)) that constitute both the heterotrimeric type IV collagen α1α1α2(IV) and the minor type IV collagens α3α4α5(IV) and α5α5α6(IV). As well as type I collagen, type IV is the most represented in the solid tumor ECM [192]. More specifically, collagen IV reorients within the ECM of various solid tumors, such as hepatocellular carcinoma, breast cancer, and colorectal cancer, conferring tissue stiffness around neoplastic nests. The peritumoral architecture, driven by the dense deposition of collagen IV and the aligned bundling of fibers resulting from the synergistic activation of proteases and lysyl oxidase, creates fibrotic matrix spots with abnormal mechanical properties. These spots attract tumor cells to migrate toward them through a process known as durotaxis [193,194,195]. This, in turn, strengthens the action of integrins and cytoskeletal interactions, which positively modulate the aggressive phenotype of tumor cells [196].

Moreover, during embryonic development, COL IV plays a crucial role in supporting the architecture of the vascular basement membrane, particularly in maintaining the structural integrity of small vessels, mediated by the non-catalytic activity of lysyl oxidase-like protein-2 (LOXL2) [197,198]. However, increased COL IV in TEM has a controversial role, as it may negatively or positively impact tumor angiogenesis [199,200].

For instance, some studies have shown that certain peptides derived from the NC1 domains of COL IV used for cancer therapy, such as Arresten (α1 chain), Canstatin (α2 chain), Tumstatin (α3 chain), and Tetrastatin (α4 chain), suppress vessel formation and cell growth in solid tumor [201,202,203]. These peptides, called collagen-derived antiangiogenic factors (CDAFs), play a critical role in inhibiting angiogenesis by interacting with specific integrin subtypes, influencing neovascularization in different ways [204,205]. Arresten’s activity is regulated by p53, as it controls both its transcription and MMP-dependent remodeling activity [206]. Moreover, the activity of Arresten and Canstatin can directly or indirectly influence the pro-angiogenic effects of VEGF or basic fibroblast growth factor [207]. Canstatin can decrease phosphatidylinositol 3-kinase/Akt signaling activity, downregulating VEGF activity, while Arresten can also sensitize endothelial cells to apoptosis by reducing the levels of the anti-apoptotic protein FADD-like IL-1β-converting enzyme)-inhibitory protein (FLIP) [208,209,210]. However, a recent study indicates that COL4A1 matrix expression and its heightened interaction with integrin beta-1 (ITGB1) in the endothelial cells of the tumor microenvironment is correlated with increased angiogenesis and chemoresistance, raising further questions about COL IV’s role in tumor angiogenesis [211].

Beyond influencing vessel development, type IV collagen is widely recognized for its involvement in various tumor processes. It is one of the most extensively studied collagens in the solid tumor ECM. For instance, in luminal breast carcinoma, COLIVα5 promotes tumor proliferation through the non-integrin receptor DDR1. Through interaction with DDR1, COLIVα5 supports the Warburg effect of tumor cells by influencing c-Myc phosphorylation and regulation of p38 Mitogen-Activated Protein Kinase (MAPK), which are essential for breast cancer progression [212]. Moreover, overexpression of type IV collagen mRNA was found not only in human breast cancer tissue but also in metastatic tissues of poor survival patients [213]. Therefore, this type of collagen could also stimulate prometastatic mechanisms. For this reason, similar to type XX, XXI, and XXII collagens, type IV collagen in the serum of patients with colorectal tumors may be a potential biomarker for liver metastases [214].

Type VIII collagen (COL8) is involved in tumor cell invasion and migration, as well as drug resistance [149]. Its expression is determined by tissue hypoxia, and it correlates with the aggressiveness of several solid tumors, as seen in MDA-MB-231 and Hs578T, two cellular subtypes of triple-negative breast cancer [215]. COLVIII regulates various signaling pathways, including the interaction between DDR1 and COL VIII, which promotes pancreatic ductal adenocarcinoma progression, through PI3K-Akt and FAK-NF-κB upregulation [216]. In this context, inhibiting COLVII expression in vitro and in vivo using lentivirus or siRNA has been found to reduce the metastatic potential and gemcitabine resistance of pancreatic ductal adenocarcinoma cells [217].

Finally, type X collagen is associated with a poor prognosis in patients with several types of solid tumors, such as breast cancer, colorectal cancer, gastric cancer, and lung adenocarcinoma [218,219,220]. In lung adenocarcinoma patients with poor prognosis, COL10A1 is upregulated and could physically interact with DDR2 to maintain a high level of FAK expression [221]. In addition, the direct involvement of COLX in epithelial–mesenchymal transition has been shown by analysis of tissues isolated from patients with aggressive forms of colorectal cancer [219].

### 7.4. Beaded Filament-Forming Collagens

Beaded filament-forming collagens are a family that includes type VI, XXVI, and XXVIII collagens. The beaded filaments appear as flexible, unbranched structures with a width of approximately 3 nm and a length of at least 2 microns [222]. Among them, type VI collagen (COL6) is a crucial component of the extracellular matrix, located in the basal membrane. Its main isoforms include the chains α1 (VI), α2 (VI), and α3 (VI). Moreover, there are three additional subunits (COL6A4, COL6A5, and COL6A6) that have been identified subsequently [223]. COL6 forms a microfilamentous network that structurally supports cells and interacts with other ECM molecules, affecting their biological functions, such as tumor response. In solid tumors, acting as a scaffold between different types of collagens and cellular receptors such as integrins, COL6 is involved in the formation of new blood vessels through the assembly of the basement membrane, a unique feature among collagens and a fundamental process in tumorigenesis. The expression levels of collagen VI vary in different cancers such as glioblastoma, ovarian cancer, and pancreatic ductal adenocarcinoma, of which different expression is probably cancer-dependent [224,225]. For example, the increased expression of COL6 is related to the aggressiveness of tumor cells, as seen in bladder cancer of high-grade T1 patients [226]. COL6, mainly produced by fibroblasts and adipocytes, supports tumor stability, and transcriptional program maintenance is related to the aggressiveness of glioblastoma cells. For this reason, its silencing in glioblastoma cells causes a downregulation of genes related to tumor progression and DNA repair mechanisms, including PI3K/AKT, Insulin-like Growth Factor 1(IGF1), Fms-like Tyrosine kinase 3 (FLT3), Platelet-Derived Growth Factor (PDGF), and MAPK [227]. In addition, pancreatic ductal adenocarcinoma cells isolated from areas of low-rigidity tumor tissue have been seen to increase COL6 expression, which has promoted in vitro motility and in vivo metastatic potential of the tumor cells [228]. Similarly, in lung sarcoma metastases, a high expression of COL6 has been detected, suggesting that it is critical for the development of metastatic niches [229].

To date, the roles of cancer of type XXVI collagen (COL26) and type XXVIII collagen (COL28) in cancer remain poorly understood. A recent study showed that in patients with poor prognosis thyroid carcinoma, there was an overexpression of the alpha 1 chain of type XXVI collagen (COL26A1). However, the signaling pathways in which it is involved remain unclear [230]. Moreover, the serum collagen levels of type XXVIII were found to be high in patients with lung cancer, but also in this case the roles are not known yet [231].

### 7.5. Anchoring Fibrils Collagens

Anchoring fibrils collagens are fibrous structures located in the lamina of different external epithelia that allow the attachment of the epidermal basement membrane to the dermal extracellular matrix [216]. Type VII collagen (COL7) is the only component of this group and its role in tumorigenesis is little understood [232]. Some studies show that hyperexpression promotes tumor progression since this type of collagen creates a scaffold between stroma and tumor epithelium [233]. However, it has been shown that its reduction during the advanced stages of the disease is associated with greater invasiveness of various solid tumors. For example, immunohistochemical studies have demonstrated that COL7A1 is highly expressed in the stroma surrounding tumor cells in solid tumors, such as moderately and poorly differentiated gastric adenocarcinomas, thereby promoting neoplastic processes [234,235]. Moreover, hypomorphic mice with reduced Col7 expression exhibited heightened tumor invasiveness in oral squamous cell carcinoma, suggesting a possible causal link. In addition, the COL7 absence in keratinocytes increases the expression of lysosomal proteases and matrix metalloproteinases, promoting cell invasion [236]. Moreover, in gastric carcinoma immunohistochemistry, it was observed that expression of type VII collagen alpha chain (COL7A1) was higher in tumor tissue than in healthy tissue, mainly localized in the extracellular matrix [234]. An increase in COL7 in the initial stages of the disease may be functional to the progression. On the other hand, its reduction during the more advanced phases can increase the invasive and metastatic potential of the cancer cells of some solid tumors, such as oral carcinoma.

### 7.6. Transmembrane Collagens

Transmembrane collagen proteins are trimers of α chains and exhibit a characteristic type II membrane protein structure. They are involved in various functions, including cell adhesion and epithelial–mesenchymal interactions [237]. This subgroup includes type XIII, XVII, XXIII, and XXV collagens. Among these, the role of type XVII collagen (COL17), a component of hemidesmosome, is particularly controversial. This is because its presence may be associated with a risk factor for patients, depending on the cancer type. In particular, the transmembrane COL17 plays a crucial role in anchoring undifferentiated keratinocytes to hemidesmosomes, while its expression is reduced in mature keratinocytes [238,239]. However, its expression significantly increases in melanomas and squamous cell carcinomas, where its deposition at the vertical fronts of the tumor is statistically associated with tumor cell invasiveness [152,153]. Moreover, a high expression of type 17 collagen alpha 1 chain (COL17A1) may be associated with a positive course for breast cancer patients, while it could be an unfavorable prognostic marker for pancreatic cancer patients [240,241]. A recent study showed how COL17 negatively affects the growth and proliferation of breast cancer cells. In particular, the tumor progression was inversely proportional to the expression of COL17, both in MCF7 and MDA-MB-231 cells and in xenotransplanted mice with overexpressed COL17 cells. COL17 inhibited the AKT/mTOR pathway and consequently reduced activation of downstream effectors such as p70S6K and 4EBP1, essential for tumor progression [242]. Furthermore, its role in colorectal cancer has also been analyzed. The absence of COL17A1 positively affected the cell cycle of dormant colorectal cancer stem cells (LGR5p27), leading to a poor prognosis for patients. More specifically, chemotherapy exposure caused a remodeling of the ECM involving COL17A1 degradation. In this way, the absence of COL17A1 promotes the FAK-YAP pathway, restoring the proliferation of dormant LGR5p27 [243]. Finally, COL17A1 can be used as a tumor biomarker, since serum COL17 levels were higher in patients with different solid tumors, including pancreatic cancer, compared to healthy controls [244].

The involvement of type XIII, type XXIII, and type XXV collagens in cancer ECM has been marginally analyzed. However, most recent studies analyze the involvement of type XIII collagen (COL13) in the invasion, progression, and migration of triple-negative breast cancer cells (MDA-MB-231). COL13 interacts with the integrin β1 and active downstream TGF-β contributing to tumor progression, as seen also in the mammosphere. In addition, COL13 boosts staminality and anoikis resistance of breast cancer cells, during the migration process [245]. Anoikis is apoptosis induced by the detachment of cells from the origin environment, through the disassembly of integrin–ECM and cadherin–cell [246]. Resistance to this mechanism plays a fundamental role in the circulating survival of cancer cells with migratory abilities [247].

Recent research has investigated the role of the α1 chain of collagen XXIII (COL23A1) in clear cell renal cell carcinoma (ccRCC). Analysis of tissues from patients with ccRCC with poor prognosis showed that this molecule enhances the progression, adhesion, and migration of cancer cells. Furthermore, knockdown underregulated the adhesion, proliferation, and migration of ccRCC cells, suggesting that COL23A1 plays a crucial role in this context [248].

Finally, the role of type XXV collagen in tumors is not well understood. There is only one study (2022) that identified for the first time the presence of alpha-1 collagen chain type XXV (Col25a1) in the ECM of murine breast tumors, while its expression was not found in the corresponding healthy tissue and human breast cancer tissue [249]. Consequently, its association with healthy and tumor ECM remains unclear.

### 7.7. Multiplexine

Multiplexins are basement membrane-associated collagens, including type XV (COL15) and type XVIII (COL18) collagens [250]. Despite the structural similarity and localization in the basal membrane, type XV and type XVIII collagens have distinct biological functions. COL15 is involved in the development of muscles and microvessels, while COL18 is in the development of the brain and eyes [251]. Their functions in tumorigenesis have been poorly understood. However, in the past decade, a possible role of COL15 has been identified as a cervical cancer suppressor [252]. Moreover, recent studies have shown its direct involvement in the suppression of hepato-carcinoma cells. More specifically, the silencing of the type XV collagen alpha chain (COL15A1), obtained by small interfering RNA, significantly enhanced the growth, invasion, and migration of hepato-carcinoma cells induced by the hepatitis B virus [253]. In addition, it has been shown that COL15 could impede hepato-carcinoma cell migration by underregulating the transcription factor Snail/Slug-mediated EMT [254].

In contrast to COL15, COL18 seems to have a positive role in the growth of solid tumors, such as in clear cell renal cell carcinoma and breast cancer [255]. COL18 interacts with the receptor tyrosine kinase of epidermal growth factor and with integrin α6 in breast cancer. The TSP-1 N-terminal domain of COL18 interacts with its receptors, triggering the downstream activation of MAPK/ERK and PI3/AKT pathways. In this way, COLXVIII supports the proliferation and migration of clear cell renal cell carcinoma [256].

Finally, the literature currently lacks the role of type II, IX, and type XXIV collagen in tumor ECM.

The various roles of specific tumor collagens are listed in Table 1.

## 8. Conclusions

Collagen is the most common protein in mammals and is the main component of the extracellular matrix. In normal tissues, a dynamic biophysical and biochemical interaction is established between collagen fibers and resident fibroblasts, where tensile and rebound energy in tissue is translated into the activation of appropriate mechanical transduction pathways [27,120,258]. Therefore, the structural changes that collagen undergoes are not only limited to the physical concept of the tissue but also modulate the biochemistry of the cells that compose it. However, these mechanisms are also critical for the development of various diseases, such as cancer [12,259].

In this context, a continuous crosstalk is established between collagen fibers within the tumor microenvironment and cancer cells. Structural modifications and variations in matrix collagens are perceived by cancer cells, activating mechanotransduction pathways, which modulate tumor progression, invasion, and migration.

For instance, type I collagen promotes epithelial–mesenchymal transition, thereby enhancing cancer cell invasion. Other collagens, such as types V, VI, VIII, and XII, facilitate tumor progression by upregulating the PI3K/Akt signaling pathway. In addition, collagen can modulate the immune system activity, contributing to tumor progression [94], acting as a physical barrier to the infiltration of the immune cells. However, some collagens can suppress these mechanisms in tumors. For instance, a reduction in type III collagen in ECM breast cancer is associated with a poor prognosis. Type XV collagen has also been identified as a tumor suppressor. This is due to its action upstream of EMT-related signaling pathways by downregulating transcription factors Snail/Slug. Furthermore, some peptide fragments derived from collagen type IV can inhibit neo-angiogenesis in tumors, with possible therapeutic applications [204].

Unfortunately, significant data on the role of type II, IX, and XXIV collagens in the extracellular matrix of tumors are still lacking in current scientific research. While other collagen types have been studied over the years, their impact on solid tumors remains uncertain. This review provides an overview of the roles of various collagen types in the extracellular matrix of solid tumors. A deeper understanding of these molecules could lead to significant advancements in the diagnosis, prognosis, and treatment of solid tumors. In recent decades, the use of collagenases in therapeutic applications—both as free enzymes and when conjugated to nanoparticles—has gained attention in the scientific community. These enzymes have the potential to break down the collagen-rich extracellular matrix of tumors, facilitating their treatment. The proteolytic activity of collagenases can improve the permeability of tumors to chemotherapeutic agents and immune cells, resulting in enhanced effectiveness of various cancer therapies. In summary, these innovative approaches represent a new frontier in the treatment of solid tumors, with collagen serving as a primary therapeutic target [260,261,262].

## Figures and Tables

**Table 1:** Collagen families, types, and their role in tumor progression.

Collagen Family	Collagen Type	Tumor	Roles	References
Fibrillar Collagen	Type I	Triple-negative breast carcinoma, pancreatic ductal adenocarcinoma, and gastric carcinoma	Increased metastasis, proliferation, and invasion.	[139,146,147,148,149,150,151,152,153,154,155]
	Type II *	Not reported	Not reported	Not reported
	Type III	Breast cancer	Reduction in invasiveness, tumor growth suppression; increased cell quiescence; inhibition of autophagy	[48,146,155,156,157,158,159]
	Type V	Gastric cancer	Increased proliferation and epithelial–mesenchymal transition	[160,161,162]
	Type XI	Papillary thyroid carcinoma, breast cancer, colorectal carcinoma, esophageal cancer, gastric cancer, pancreatic cancer, lung cancer, and ovarian cancer	Promotion of proliferation, drug resistance, and tumor invasiveness	[63,163,164,165,166,167,168,169,170,171,172,173,174]
	Type XXVII	Melanoma cells	Promotion of tumor cells quiescence	[175]
	Type XXIV *	Not reported	Not reported	Not reported
FACITs	Type XII	Breast cancer, colorectal cancer, pancreatic adenocarcinoma, and ovarian cancer	Promotion of tumor progression and metastasis	[179,180,181,182,183]
	Type XVI	Colorectal cancer (CRC), glioblastoma, and oral squamous cell carcinoma	Increased invasiveness	[184,185,186]
	Type XIV	Melanoma	Reduction in malignancy	[187]
	Type XIX	Melanoma	Inhibition of tumor cell migration and invasion	[188]
	Type XX	Pancreatic ductal adenocarcinoma	Not reported	[189]
	Type XXII	Pancreatic ductal adenocarcinoma	Not reported	[189]
	Type XXI	Gastric carcinoma	Increased tumor progression	[190]
	Type IX *	Not reported	Not reported	Not reported
Network-forming collagen	Type IV	Breast carcinoma, colorectal tumor, hepatocellular carcinoma	Promotion of tumor proliferation, durotaxis, angiogenesis, and metastasis. Reduction in angiogenesis	[192,193,194,195,196,197,198,199,200,201,202,203,204,205,206,207,208,209,210,211,212,213,214]
	Type VIII	Breast and pancreatic ductal adenocarcinoma	Promotion of tumor cell invasion and migration, as well as drug resistance	[149,215,257]
	Type X	Breast cancer, colorectal cancer, gastric cancer, and lung adenocarcinoma	Promotion of tumor cell proliferation, migration, and invasion	[218,219,220,221]
Beaded filament-forming collagen	Type VI	Glioblastoma, ovarian cancer, and pancreatic ductal adenocarcinoma	Promotion of metastasis, angiogenesis, and tumor progression	[224,225,226,227,228,229]
	Type XXVI	Thyroid carcinoma	Not reported	[230]
	Type XXVIII	Lung cancer	Not reported	[231]
Anchoring fibril	Type VII	Oral squamous cell carcinoma and gastric carcinoma	Promotion of tumor progression; block invasiveness	[232,233,234,235,236]
Transmembrane collagen	Type XVII	Squamous cell carcinoma; breast cancer, colorectal cancer	Promotion of cell migration and invasion. Block tumor progression	[238,239,240,241,242,243,244,245,246]
	Type XIII	Breast, clear cell renal cell carcinoma, and prostate cancer	Tumor progression, invasion, resistance to anoikis, and tumor metastasis	[245,246,247]
	Type XXIII	Clear cell renal cell carcinoma	Promotion of tumor progression, adhesion, and migration	[248]
	Type XXV	Breast cancer	Not reported	[249]
Multiplexin	Type XV	Cervical cancer; hepato-carcinoma	Reduction in growth, invasion, and migration	[251,252,253,254]
	Type XVIII	Clear cell renal carcinoma, breast cancer	Increased proliferation and migration	[252,256,257]

* Roles of collagens II, XXIV, and IX in tumor progression uncleared.

## Data Availability

Not applicable.
